# A randomised feasibility study assessing an intervention to keep adults physically active after falls management exercise programmes end

**DOI:** 10.1186/s40814-020-00570-9

**Published:** 2020-03-07

**Authors:** Sarah Audsley, Denise Kendrick, Pip Logan, Matthew Jones, Elizabeth Orton

**Affiliations:** 1 Division of Primary Care, University of Nottingham, Nottingham, NG7 2RD UK; 2 Division of Rehabilitation, Ageing and Wellbeing, University of Nottingham, Nottingham, UK

**Keywords:** Older adults, Physical activity, Falls prevention, Feasibility study

## Abstract

**Background:**

Physical inactivity contributes to disability and falls in older adults. Falls prevention exercise (FaME) programmes improve physical activity and physical function and reduce falling rates. Improvements in physical function are reduced, and falls rates increase, if physical activity is not maintained. This research investigated the feasibility and acceptability of an intervention that aimed to maintain physical activity in older adults exiting FaME.

**Methods:**

The Keeping Adults Physically Active (KAPA) intervention comprised of six group sessions of motivational interviewing, delivered monthly by trained and mentor-supported postural stability instructor’s after the FaME programme ceased. The KAPA intervention included participant manuals, illustrated exercise books, physical activity diaries and pedometers. A feasibility study was conducted in 8 FaME classes. The study design was a two-arm, cluster randomised, multi-site feasibility study comparing the KAPA intervention with usual care. A sample of 50 community-dwelling adults aged 65 years old or older were recruited. Recruitment, retention and attendance rates, self-reported physical activity and participant interviews were used to examine the feasibility and acceptability of the KAPA intervention.

**Results:**

Fifty of the sixty-seven (74.6%) participants invited into the study agreed to take part, 94.2% of the available KAPA sessions were attended and 92.3% of the recruited participants provided outcome data. The KAPA participants expressed positive views about the venues and postural stability instructors and reported enjoying the group interactions. Intervention participants discussed increasing their physical activity in response to the peer-support, illustrated home exercise booklet, physical activity diaries and pedometers. Most discussed the written tasks to be the least enjoyable element of the KAPA intervention. The proportion of participants reporting at least 150 minutes of moderate to vigorous physical activity per week rose from 56.3 to 62.5% in the intervention arm and from 41.4 to 52.0% in the usual care arm.

**Conclusions:**

The participants found the KAPA intervention acceptable. Participants reported the exercise booklet, peer support and the physical activity monitoring tools encouraged them to keep active. A full-scale trial is needed to assess whether physical activity can be significantly maintained in response to the KAPA intervention.

**Trial registration:**

Retrospectively registered on ClinicalTrials.gov (NCT03824015).

## Introduction

Approximately 33% of adults aged over 65 years, and 50% of adults aged over 80 experience falls each year, with 20% resulting in injury [1–4]. The National Health Service is estimated to spend £2.3 billion per year treating falls [5]. The human cost of falls includes injury, declines in physical function and loss of confidence and independence [3,4].

Physical inactivity in older adults results in muscle weakness, poor balance, functional impairment and an increased falls risk [6,7]. Exercise improves muscle strength, balance and physical function and reduces falls rates in older adults by 23% [8–11]. Therefore, clinical guidelines recommend older adults at risk of falls attend falls prevention exercise programmes [12]. The Falls Management Exercise (FaME) programme contains age-specific strength, balance, cardiovascular and flexibility exercise aiming to improve physical function and reduce falls risk in older adults. The ProAct 65+ trial showed FaME significantly increased moderate to vigorous physical activity (MVPA) and reduced falls rates in FaME attendees at 12 months, but not at 24 months [13].

The current study developed a multicomponent behaviour change intervention called the Keeping Adults Physically Active (KAPA) programme to encourage the continuation of physical activity (PA) in older adults exiting FaME programmes. Our research aim was to investigate the feasibility and acceptability of the KAPA intervention to the participants. To meet this aim, research objectives investigated attendance and adherence rates to the KAPA programme and evaluated its acceptability via semi-structured interviews and questionnaires. Parameter estimates of MVPA, recruitment and retention rates, research costs, programme fidelity and adverse events were investigated to inform the design of a definitive trial.

## Methods

### Study design

This was a mixed-methods, two-arm, (multisite) cluster randomised feasibility study comparing the KAPA programme with usual care.

### Setting

Participants were recruited from government-commissioned-based FaME classes, delivered in the community by leisure service providers within Derby City, Rutland and Leicestershire Counties.

### Participants

There were two types of participants:

1. Community-dwelling FaME programme service users aged 65 years or older.
2. Postural Stability Instructors (PSIs) delivering FaME classes.

All PSIs and FaME class attendees were considered eligible. Participants were ineligible if unable to provide written consent. Participants were withdrawn from the study at their request or if they could not safely engage with PA.

### Sample size

A sample size calculation informed the recruitment targets [14]. We anticipated there would be 12 PSIs, each delivering one FaME class containing 10 participants. With 12 PSIs and 120 participants in 12 classes, and an intra-class coefficient (ICC) of 0.05 [15]; the feasibility study would be able to detect a recruitment rate of 70% with a 95% confidence interval (CI) ranging from 60 to 80%.

### Recruitment

Initial recruitment was poor so two recruitment periods were conducted to increase recruitment rates (recruitment strategy 1: January to February 2017 and recruitment strategy 2: June to July 2017). Researchers e-mailed eligible PSIs to provide study information and request their participation. Recruitment strategy 1 took place between weeks 20 and 24 of the original FaME classes. Each recruited PSI provided oral and written study information and an expression of interest slip to the FaME class attendees during their classes. Recruitment strategy 2 took place up to 6 months after the original FaME class completion. Each recruited PSI provided written study information and an expression of interest slip to the participants either during the usual care classes or by phone and post. Researchers received expression of interest slips and met with interested participants to answer questions and obtain written consent.

### Randomisation

PSIs and participants were recruited prior to randomisation. PSIs were randomised in a 1:1 allocation ratio to deliver KAPA or usual care. PSIs were stratified by study centre (2 strata) and randomly allocated within strata. The study statistician computer generated the random allocation. Two PSIs delivering the same FaME class were allocated as one unit. Some PSIs delivered two FaME classes, therefore one class was selected by an independent researcher tossing a coin.

### Allocation concealment

Study arm allocations were placed in numbered sealed opaque envelopes and grouped by stratum. PSIs were chronologically numbered depending on their recruitment date. An independent researcher opened the envelopes and documented the PSI’s allocation. PSIs and participants were blinded to the group allocation until all participants were recruited. Researchers were not blinded.

### Intervention procedures

#### KAPA training and mentoring programme procedures

Intervention arm PSIs were trained in motivational interviewing, the KAPA programme and were given a trainee handbook and standard operating procedures. PSIs received up to three, 1-hour, mentoring sessions to support the effective delivery of KAPA.

#### KAPA intervention arm procedures

Participants received six sessions of motivational interviewing and behaviour change techniques (BCTs) that aimed to motivate them to keep active. Sessions were delivered within community venues in a group setting by the PSIs. Sessions lasted between 60 and 90 minutes and were delivered over a 6-month period. Participants received a pedometer and a participant manual containing illustrated exercises, worksheets and PA diaries. KAPA was delivered by telephone if a participant was unable to attend sessions. Intervention participants had access to usual care. Table 1 outlines the intervention strategies delivered in each KAPA session.

**Table 1:** Table outlining the intervention strategies delivered in each KAPA session

Month	Session content
1	Initial consultation
Review current health	
Explore knowledge on PA and educate on the PA guidelines	
Reflect and compare current PA levels with PA guidelines	
Cost-benefit analysis and mental imagery of two alternate futures	
Provide information about local PA services	
Introduce and demonstrate illustrated exercise booklet	
Plan weekly physical activities	
Barriers and facilitators of completing the PA plan	
Identify people who can provide social support	
PA goal setting	
Rate commitment and confidence ratings towards meeting goals	
Provide and discuss the use of PA diaries and pedometers	
Document a signature of commitment	
2	Follow-up session 1
Reflect on PA diaries and goal achievement and adapt plans and goals accordingly.	
Problem solve high-risk situations and write “if then” plans.	
Encourage the use of self-monitoring tools and accessing social support	
3	Follow-up session 2:
Reflect on PA diaries, goal achievement, if then plans, and adapt plans and goals accordingly.	
Introduce relapse prevention strategies (i.e. monitoring tools, reflecting on past successes, recovering from lapses, planning coping strategies).	
Building new habits (i.e. building knowledge of habit formation, discussing poor PA habits, keeping a habit diary)	
Rewarding good PA behaviours	
Reflecting on enjoyment gained from being more active	
4 & 5	Follow-up session 3 & 4
Reflect on PA diaries, goal achievement, if then plans, and adapt plans and goals accordingly.	
Identifying and planning for possible changes in life circumstances	
Identifying plans to over-ride old PA habits	
Discussing stress management	
Planning for mentally challenging times	
6	Follow up session 5
Reflect on PA diaries, goal achievement, if then plans, and adapt plans and goals accordingly.	
Reflect on self-regulation skills	
Plan how people intend to keep physically active after KAPAs end	
Reflect on achievements and give praise	
Sign a pledge of commitment	

Each component was delivered using a motivational interviewing approach

#### Usual care arm procedures

Participants finishing the 24-week commissioned FaME programme were offered usual-care which was a weekly, self-funded, FaME exercise class.

### Data collection and outcome measures

Quantitative data were used to evaluate recruitment, retention, adherence and attendance rates, measures of fidelity, intervention cost, adverse events and PA estimates. Qualitative data were used to evaluate the KAPA programme acceptability. Service providers routinely collected the participants’ socio-demographic characteristics and the participants consented to share their data with the researchers.

**Fig. 1:**
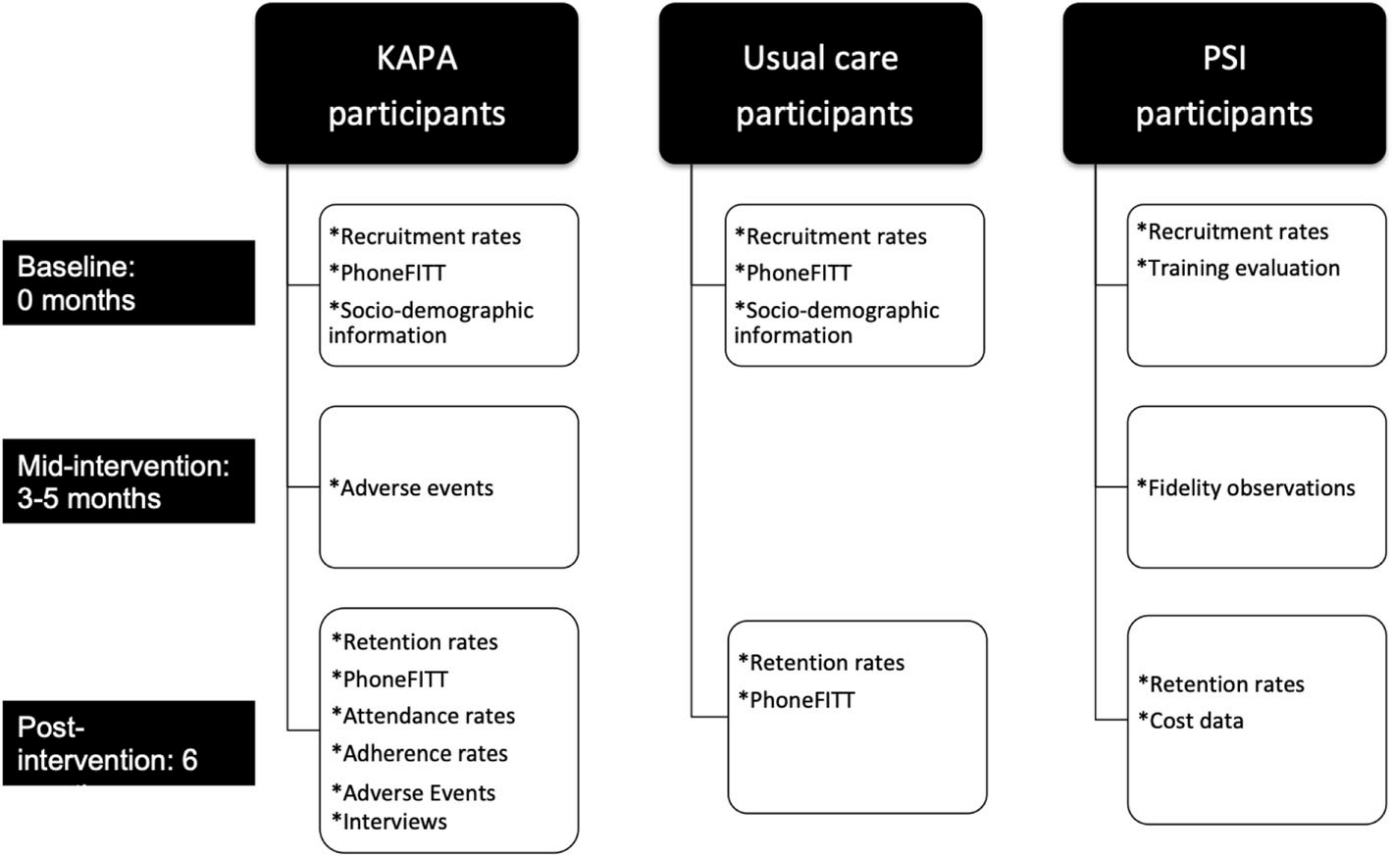
Data collection time points and data collected per study arm. PhoneFITT is a self-reported PA questionnaire

Baseline research data were collected between January 2017 and July 2017 and post-intervention data between July 2017 and February 2018. All research data were collected at the study sites. Figure 1 depicts the data collection time points and data collected per study arm.

### Recruitment and retention rates

Researchers recorded the number of PSIs and FaME class attendees invited and recruited into the study. Retention rates were recorded as the number of participants remaining in the study at the 6-month time point.

#### Measures of feasibility: KAPA participants

The PSIs documented attendance at each telephone or face-to-face KAPA session in the class registers. Self-monitoring PA and setting goals were key intervention BCTs and used to measure intervention adherence. The PSIs asked the participants if they had completed their diaries and achieved their goals during each session and recorded the responses in the class registers.

Estimates of MVPA were collected using the Phone-FITT questionnaire. Phone-FITT measures frequency, duration, intensity and type of PA performed. Phone-FITT’s reliability and validity has been established by comparing older adults’ Phone-FITT scores and accelerometer counts (Spearman’s correlation coefficient (95% CI), ranging from 0.29 (0.01, 0.53) to 0.57 (0.34, 0.73) [16]. The PSIs handed Phone-FITT questionnaires to the participants to self-complete and collected them back at the end of the KAPA/usual care sessions. Participants who stopped attending sessions were posted the questionnaire and a self-addressed envelope.

#### Measures of feasibility: PSIs

The trainer handed training evaluation forms to each PSI after the KAPA training to assess its acceptability. Evaluation forms contained questions and response options on 4 and 5-point Likert rating scales relating to the quality and usefulness of the training materials and whether the PSIs were confident to deliver KAPA.

Fidelity of the KAPA programme was ascertained by observations of one initial and follow up session per PSI. Observations lasted the class duration and were recorded on a fidelity checklist investigating the following:

- Health, safety and environment management
- Communication
- Conducting a behaviour analysis
- Reviewing behaviours
- Delivering BCTs
- Introducing and closing sessions

The observer dichotomised items as being achieved or not achieved. PSI adherence with the data collection protocol was measured by whether the class registers were fully complete.

### Cost data

A local government perspective of costing the KAPA programme was used to assess the direct costs incurred. Cost proformas were emailed to, and completed by, the service providers at the 6-month time point. Cost data included staff salaries, staff travel expenses, administration costs, venue hire, consumables and training day attendee expenses (price year 2017). A researcher recorded the costs incurred to deliver the KAPA training day (venue hire, training manuals, trainer salary and travel expenses).

### Adverse events

A researcher explained the definition of an adverse event to each intervention participant. Participants were given a contact details card and asked to contact the researcher if any adverse event occurred. A researcher telephoned the participants every 3 months to collect data.

### Interviews

All intervention arm participants were invited to a semi-structured interview. Interviews explored their perceptions on the acceptability, benefits and disadvantages of the KAPA programme. Interviews lasted up to 60 minutes and took place at the participants’ homes or a community location.

### Data analysis

Recruitment, retention, adherence and attendance rates and the training evaluation responses were described using numbers and percentages. Fidelity scores were summed as the total number and percentage of items achieved within each, and across all, criteria.

The median (interquartile range (IQR)) total weekly MVPA and the proportion meeting the government-recommended 150 minutes of MVPA per week target were estimated at baseline. The MVPA data were not normally distributed, therefore participants were dichotomised into the proportion undertaking the ≥150 minutes MVPA target. The proportion meeting the MVPA target was compared between study arms using random-effects logistic regression models to estimate the odds ratio and 95% confidence interval and adjusting for study site and baseline MVPA. A one-way analysis of variance was used to estimate the intraclass correlation coefficient for the proportion meeting the MVPA target, as described by Ridout et al. [17] Baseline data for 4 intervention and 1 usual care participants was missing and not included in any analysis.

Total staff costings over the 6-month period were calculated by the hourly staff salary multiplied by the length of time taken on intervention delivery, travel or administration. Non-salary related costs were summed and multiplied over the intervention period. Consumable costs were summed as one-off costs. The mean cost across all study sites and per participant was estimated and described.

Adverse events were summed and described narratively. Semi-structured interviews were audio-recorded and transcribed verbatim. Transcribed data were handled using NIVO10 software and coded. All interviews were analysed using a framework analysis approach [18].

## Results

### Recruitment and retention rates

Ten PSIs were eligible, including two pairs of PSIs that jointly delivered FaME classes. Ten PSIs and the attendees of eight FaME classes were recruited. Sixty-seven participants within 8 classes were invited into the study and 50 participants (74.6%) were recruited (*n* = 20 intervention arm, *n* = 30 usual care arm).

**Fig. 2:**
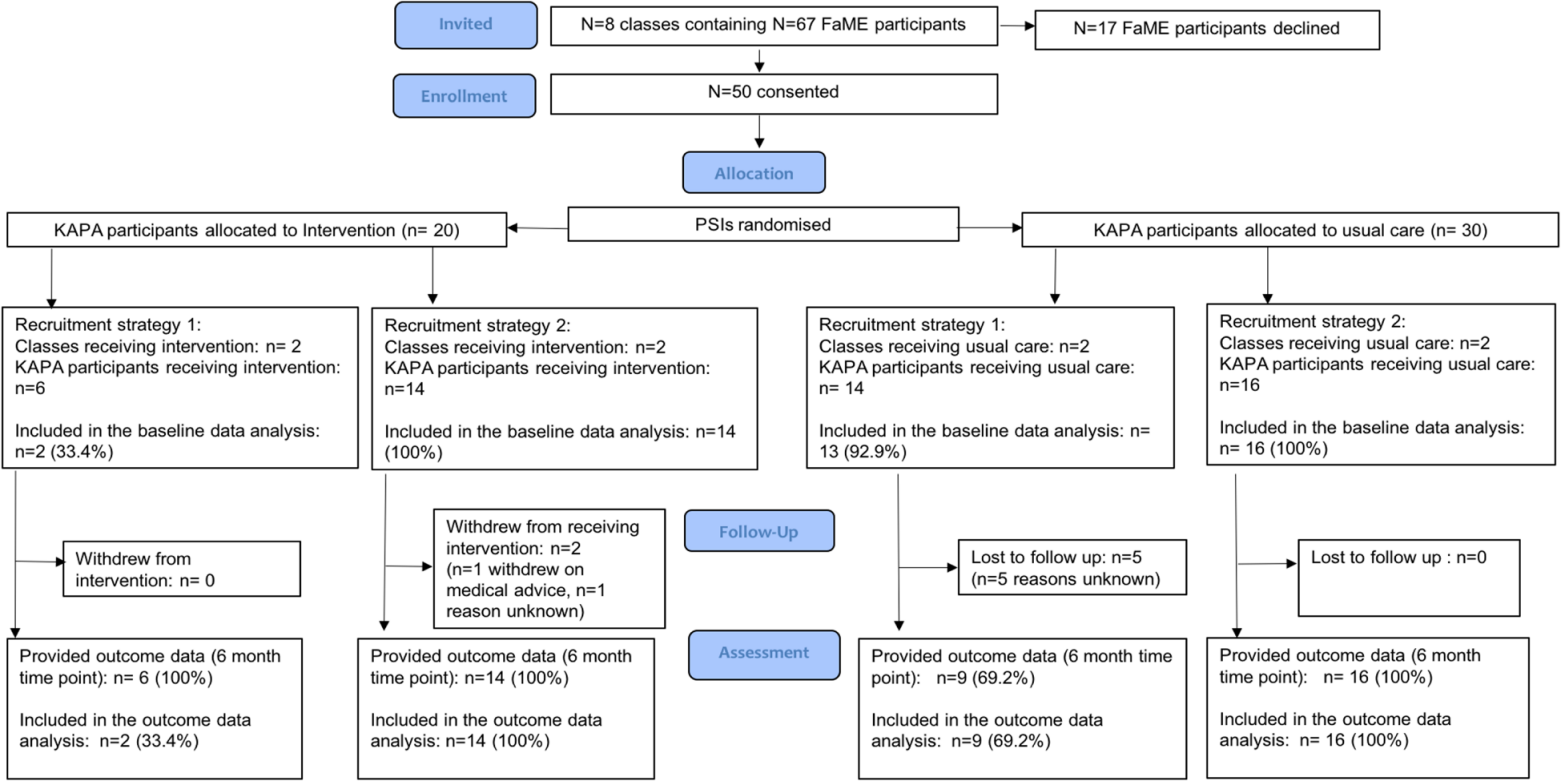
KAPA participant flow through the KAPA feasibility study

Twenty-five (83%) usual care participants provided outcome data at the 6-month time point as five participants were lost to follow-up (reasons unknown). No intervention participants were lost to follow-up and all provided outcome data. Two intervention participants stopped receiving KAPA, one based on GP advice and one whose reasons were unknown. Five (83%) intervention PSIs and four usual care PSIs provided outcome data. Figure 2 depicts the KAPA participant recruitment flow through the study.

### Sociodemographic information

Table 2 outlines the participants’ sociodemographic characteristics and PA levels at baseline.

**Table 2:** KAPA participants’ socio-demographic and baseline PA information

	Intervention arm	Usual care arm
	*n* = 16	*n* = 29
Age		
Mean, (SD)	76.9 (7.0)	73.8 (6.4)
Gender *n* (%)		
Female	13 (81.3)	20 (69.0)
Male	3 (18.7)	9 (31.0)
Ethnicity *n* (%)		
White British	15 (93.8)	29 (100)
Asian Indian	1 (6.3)	0 (0.0)
Co-morbidities *n* (%)		
0 (None)	1 (6.3)	5 (17.2)
1	2 (12.5)	7 (24.1)
2	6 (37.5)	7 (24.1)
3	2 (12.5)	5 (17.2)
4	2 (12.5)	3 (10.4)
5	1 (6.3)	2 (6.9)
6 or more	2 (12.5)	0 (0.0)
IMD *n* (%)	(n = 12)	(n = 22)
Quintile 1—most deprived	0 (0.0)	3 (13.6)
Quintile 2	2 (16.7)	7 (31.8)
Quintile 3	2 (16.7)	1 (4.6)
Quintile 4	1 (8.3)	2 (9.1)
Quintile 5—least deprived	7 (58.3)	9 (40.9)
Education *n* (%)		
Secondary school (age 15/16)	10 (62.5)	20 (69.0)
Secondary school (age 17/18)	2 (12.5)	0 (0.0)
College	1 (6.3)	6 (20.7)
University	3 (18.8)	3 (10.3)
FRAT score *n* (%)		(*n* = 28)
0	5 (31.3)	9 (32.1)
1	5 (31.3)	7 (25.0)
2	1 (6.3)	6 (21.4)
3	5 (31.3)	6 (21.4)
Physical activity		
Total minutes of MVPA—median, (IQR)	160.0 (57.5 to 532.5)	46.0 (0.0 to 267.0)
Total MVPA minutes:		
0–149 minutes MVPA n, (%)	7 (43.8)	17 (58.6)
≥ 150 minutes MVPA n, (%)	9 (56.3)	12 (41.4)

Missing baseline data 5 participants, *n* = 4 intervention arm, *n* = 1 usual care arm

*IMD* index of multiple deprivation, *FRAT* Falls Risk Assessment Tool

### Attendance rates

**Fig. 3:**
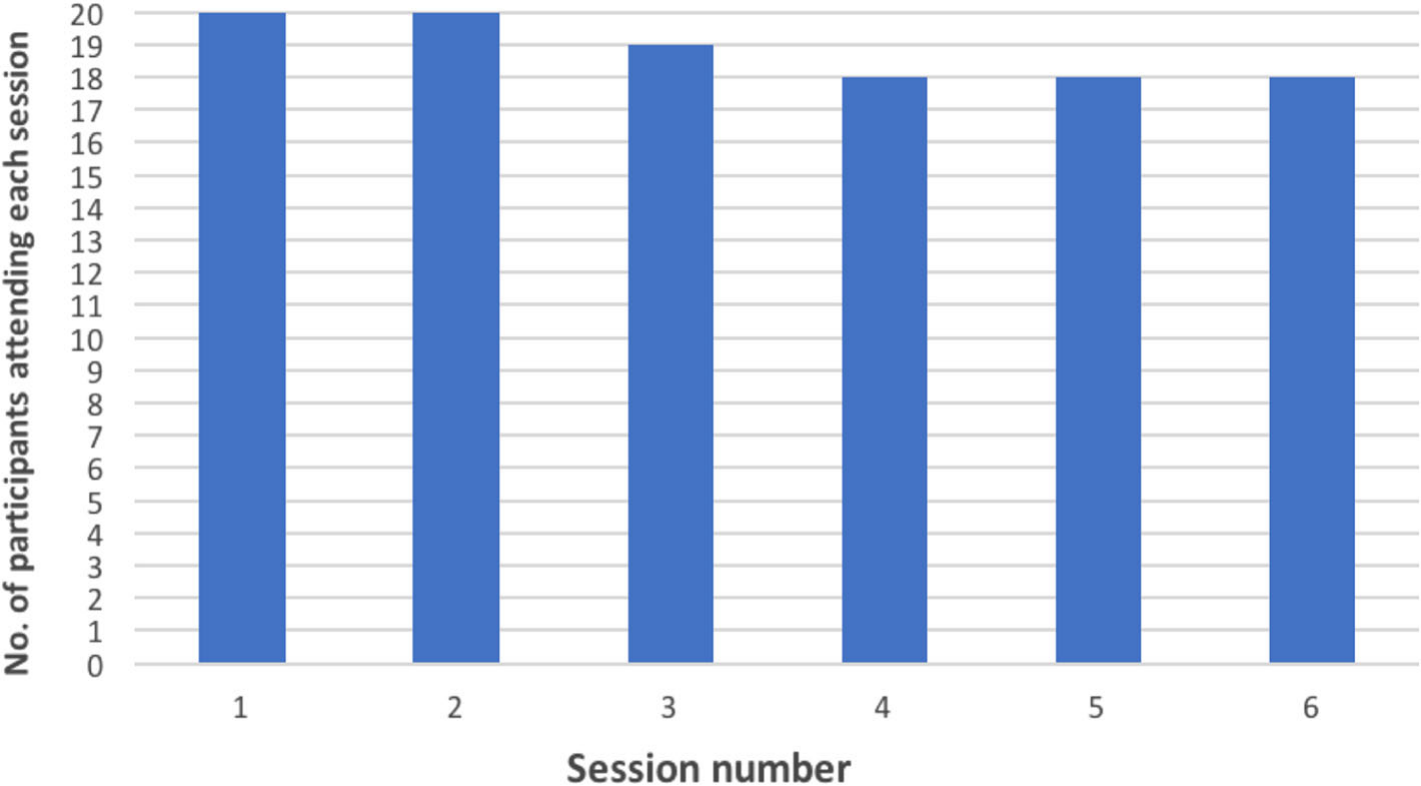
The number of participants attending each of the six KAPA intervention sessions

Twenty intervention participants attended 94.2% (*n* = 113) of the six available KAPA sessions (*n* = 120). Ninety-seven of 113 sessions were attended face-to-face (78.2%) and sixteen were attended by phone (21.8%). Figure 3 depicts the number of participants attending each of the six sessions.

### Adherence

Four participants were 100% compliant with completing all PA diaries. PA diary adherence data was incomplete for 16 participants (totalling 35% missing data). Goal attainment data was missing for 17 participants (totalling 37% missing data); the 3 participants with complete data reported achieving 80% of their PA goals.

### Estimates of MVPA

The proportion of participants achieving the MVPA target was 56.3% in the intervention arm and 41.4% in the usual care arm at baseline. Six-month follow-up data showed the proportion meeting the MVPA target rose in the intervention (62.5%) and usual care (52.0%) arms. The odds of reporting meeting the MVPA target were 25% higher in the intervention than usual care arm, but this did not reach statistical significance (OR 1.25, 95% CI 0.26 to 5.88; *p* = 0.78). The ICC for reaching the target MVPA minutes was < 0.001 (95% CI 0.00 to 0.24) Table 3.

**Table 3:** The proportion of participants and adjusted odds ratios for achieving 150 minutes of MVPA by study arm

6-month time point	Intervention arm (n = 16) *n* (%)	Usual care arm (*n* = 25) *n* (%)	OR	95% CI	*p* value
Total MVPA minutes					
0–149 minutes MVPA	6 (37.5)	12 (48.0)	Ref		
≥150 minutes MVPA	10 (62.5)	13 (52.0)	1.25	0.26 to 5.88	0.78

Missing values *n* = 4 intervention group; *n* = 5 usual care group

Adjusted for baseline value

### Training evaluation

PSIs reported either “comprehensive” or “adequate” coverage of the theory and practical skills needed to deliver KAPA. All 6 PSIs assigned to delivering the KAPA intervention reported the training and lectures were easy to follow. Three PSIs “agreed” that the learning materials were helpful. All PSIs were “confident” or “very confident” in developing PA plans, reviewing goals and delivering the follow-up sessions. PSIs reported being “confident” or “quite confident” in delivering motivational interviewing and behavioural assessments.

### Fidelity observations

Total fidelity scores achieved across all PSIs remained similar between observation 1 (74%) and 2 (75%). Between the first and second observations, PSIs’ communication scores (85 to 100%) and session closing scores (70 to 93%) improved. Scores relating to introducing the session (88% vs 65%) and delivering BCTs (67% vs 43%) reduced. Setting up the environment and health and safety scores were 100% in all observations.

### KAPA study costs

Total cost of delivering the KAPA intervention was £3,987.85 (GBP) and per participant cost was £199.39. Table 4 shows the total KAPA intervention costs incurred.

**Table 4:** Total costs for delivering the KAPA intervention

Service provider costing		
Category	KAPA intervention (*n* = 20) No. of sessions delivered across the 4 intervention sites: 52	
	Total cost (£)	Average cost per item per site (£)
Setup costs: KAPA training		
Attending KAPA training and mentoring sessions (PSI wages)	835.90	208.98
Travel expenses (PSI)	18.00	4.50
Training manuals	50.58	12.65
Venue hire	140.00	35.00
Trainer wage	420.00	105.00
Travel expenses (trainer)	32.40	8.10
Attending KAPA training and mentoring sessions (PSI wages)	835.90	208.98
Travel expenses (PSI)	18.00	4.50
Reoccuring delivery costs		
Staff salary		
Intervention delivery time (face to face and by phone)	1208.34	302.09
Administration time	386.82	96.71
Travelling time to classes	171.84	42.96
Travel expenses (i.e. petrol and parking)	54.00	13.50
Venue hire	345.00	86.25
Refreshments	30.00	7.50
Administration consumables	2.24	0.56
Telephone usage	94.45	23.61
Pedometers and participant manuals	198.28	49.57
Total intervention cost	£3,987.85	
Total per participant	£199.39	

### Adverse events

One adverse event and two adverse reactions were reported. Table 5 summarises all reported adverse events.

**Table 5:** Description of adverse events

	Description	Place of event	Adverse event category		
		During KAPA activities	Adverse event	Adverse reaction	Relation to KAPA
1.	Walking injury. Knee osteoarthritis exacerbation.	Yes	No	Yes	Possibly
2.	Fall in exercise class. Skin graze.	Yes	Yes	No	Definitely
3.	Walking injury. Plantar-fasciitis.	Yes	No	Yes	Possibly

### Qualitative data

Semi-structured interviews were conducted with 16 intervention participants. Emerging themes related to the acceptability of the PA diaries, participant manuals and intervention dosage, satisfaction with the venues and PSIs and the perceived benefits and disadvantages of taking part in the KAPA programme. Table 6 contains quotes supporting the qualitative findings.

**Table 6:** Quotes supporting the qualitative findings

Acceptability of completing the diaries	
“Well there were odd days obviously when I was out, but it got done on a daily basis it never got left until the next day” PT03_Female, 69_site 1 “The big thing I found the most helpful was the physical activity diaries, because you could take a look at the end of the week, it felt good that I had done better last week than the week before...” PT03_Female, 69_site 1 “I thought it was a bit of a pain filling in you know the sort of diary” PT02_Female, 76_site 1 “Oh yes, I will continue the book because like I say I think it focuses your mind on how many steps you’re doing and exactly what you’re doing or not doing.” PT08_Female, 67_site 3 “The only disadvantage is one doesn’t like filling in forms but that was really all.” PT12_Male, 85_site 4	
Acceptability of the participant manuals	
“The manual was very good, very instructive” PT02_Female, 76_site 1 “I found it useful that we got the exercise programme printed out for us, and when you come to do your exercises at home then you have got something to remind you” PT10_Female, 79_ site 3 “It seemed over complicated... but to have it simplified we thought.” PT11_Male, 73_site 4	
Acceptability of the intervention duration, frequency and timings	
“I think a month is about right, weekly but weekly would be a chore” PT07_Female, 84_site 3 “They finish at 1, you sort of want to get back to have a meal really …” PT01_Male, 82_site 1	
Satisfaction with the venues and PSIs	
“Well I think it is a good facility, you know everything is there that you need. The space, it is clean, there is toilets, coffee and nice people … just everything about it is just right.” PT11_Male, 73_site 4 “Well you have got a very good instructor, I think he did a good job... if you came up with a problem, he suggested how you could get around it.” PT01_Male, 82_site 1	
*Social benefits*	
“Being a group. Yes. And the fact that we see one person is doing something, and if another one is ill and can’t do it, we are there encouraging them that they are doing the best they are able to within their abilities. So, they don’t feel that they are not achieving anything because we are encouraging them.” PT07_Female, 83_site 3 “Because one of the important features of those classes was the social interaction with other people.” PT12_Male, 85_site 4	

#### Acceptability of completing the diaries and pedometers

Diary completers believed the diaries focused their mind on their goals and they felt motivated and satisfied by their achievements. Participants who found completing the diaries inconvenient discussed finding the pedometer motivating. A number of participants reported they were still using the pedometers and diaries to motivate them to keep active after KAPA ended.

#### Acceptability of the participant manuals

Participants expressed a variety of views on the acceptability of the participant manuals. Numerous participants found the manuals informative and worksheets useful. Yet others found the worksheets repetitive and over complicated and suggested they would be improved if “simplified”. All participants believed the illustrated home exercise booklet helped “remind” them how to perform the home exercises.

#### Acceptability of the intervention duration, frequency and timings

Most participants felt the intervention duration was “long enough” and the frequency of sessions was “about right” and they would not have benefited from a longer intervention period or more frequent sessions. Running the KAPA programme over a lunchtime was a barrier as many participants wanted to *“get back”* home for a meal.

#### Satisfaction with the venues and PSIs

Participants were highly satisfied with the venues as they were accessible and had good parking and refreshment facilities. Participants positively viewed the PSIs personal characteristics and found them motivating, encouraging, helpful and knowledgeable.

#### Social benefits

The KAPA programme’s main benefit from the view of the participants was the enjoyment gained from interacting with others in a group. Participants discussed feelings of encouragement and motivation from their group and how they enjoyed encouraging others. Participants who lived alone particularly found these opportunities helpful.

## Discussion

### Summary of findings

The KAPA intervention was feasible to deliver within community-based PA services, and acceptable to older people exiting FaME classes. Due to small class sizes and PSI numbers the target of recruiting 120 participants was not achieved. Ninety percent of participants received the KAPA programme until the 6-month time point and attendance rates were high (94%), suggesting it was feasible to attend. Participant adherence with the PA diaries and goals could not be ascertained due to missing data. Fidelity results suggest that PSIs communicated well but many BCTs were not delivered. A higher proportion of the intervention arm reported achieving the MVPA target, but this did not reach statistical significance.

### Interpretation of the findings

PA studies in older people often do not recruit enough participants to meet sample size requirements [19–26]. Only one FaME class per PSI was selected which limited the number of classes and participants reached. More PSIs need to be recruited to allow for more classes and participants to be recruited in a definitive trial.

Social activities give older adults enjoyment and the motivation to exercise [25, 27–31]. Similarly, the KAPA participants reported enjoying interacting with their peers, which motivated them to attend the KAPA sessions. Therefore, fostering social networks may be an integral component in interventions aimed at older adults. Substantial evidence shows that older adults remain active in response to self-monitoring and goal-setting activities [20, 21, 24–26, 32–44]. Comparably, KAPA participants often spoke of the motivation gained by meeting their goals and monitoring their activity. Similar to other PA studies [25, 40], we had a mixed reception to the manual and writing activities whereby some participants found them helpful and others did not. Thus, writing activities may need to be tailored to individual participants to improve programme acceptability.

Authors investigating PA maintenance programmes report an average of 20% of participants experience adverse events [25, 40]. Our 15% adverse event rate is similar to other studies, suggesting the KAPA intervention does not require major modification to reduce potential harm.

### Strengths and Limitations

Retention and attendance rates were high showing that the KAPA intervention has good potential to retain the participant numbers needed to maintain statistical power in a definitive trial. We aimed to assess intervention adherence by measuring the proportion of people completing the PA diaries and achieving their PA goals. However, we were unable to draw conclusions about adherence due to a substantial amount of missing data. It is unknown why such a large amount of data was missing. To help overcome data collection deficiencies a definitive trial would benefit from the research team collecting the adherence data.

The point estimate of the KAPA intervention potential treatment effect (maintenance of PA) remains unknown as seven of the eight study sites were offered self-funded FaME classes as part of usual care. This likely contributed to MVPA levels being maintained. To reduce the possibility of type 2 errors future trials assessing the effects of maintenance interventions should avoid such contamination.

The Phone-FITT questionnaire is a validated tool to collect self-reported PA data using an interview approach [16]. The KAPA feasibility study did not use the tool in its validated form as the study participants self-completed the Phone-FITT questionnaire, and it is unclear whether using a none interview approach affected its validity or reliability of the PA outcomes [45]. Therefore a definitive trial should collect Phone-FITT data using an interview approach and quantify the results using validated methods [16].

The recruitment period was extended, resulting in a 6-month intervention delivery delay to many participants. Treatment effects of exercise interventions (such as FaME) are known to diminish after the intervention is discontinued [33, 46]. Therefore, the absolute increases in MVPA may be greater in the participants receiving a delayed intervention as PA may have reduced after FaMEs end and later spiked in response to KAPA. In a definitive trial, all participants should receive the KAPA intervention within the same time window and immediately after the end of the FaME classes to reduce baseline PA differences and maximise potential effects.

### Generalisability

In the UK, FaME classes are provided within many different settings including healthcare settings [47]. Whilst we know that the FaME programme is effective regardless of setting, we do not know whether FaME and KAPA being delivered in a leisure service setting increase the likelihood of maintaining PA. Thus, the study results cannot be generalised into classes being delivered within healthcare settings. KAPA participants selected themselves into FAME classes and into KAPA. Therefore, it is possible that the people needing KAPA the most were not reached. To help improve the study findings generalizability, future trials should aim to recruit older adults from a wider geographical area and diversity of settings.

### Implications for progression to a definitive trial

A definitive trial should anticipate class and participant numbers based on our findings and should aim to recruit more study sites. Using PSIs to collect adherence data was an ineffective strategy. Yet, diary return rates to researchers are 50 to 60% in older adults [48]. A possible solution could be for PSIs to collect adherence sheets during the KAPA sessions to forward to the research team.

Fidelity results show the PSIs did not deliver the BCTs as intended. Training evaluation outcomes suggest some PSIs were only “quite confident” in delivering the BCTs which may have affected fidelity. Fidelity improves when monitoring and feedback loops are built into interventions [49–51]. A definitive trial could improve fidelity by feeding back provider performance for training purposes.

Accurately measuring PA is important to assess intervention effectiveness [45]. Self-reported PA is less reliable compared with objectively measured PA [45]. Especially in older adults who are known to overestimate MVPA as a result of poor PA recall and social desirability bias [44,52,45]. MVPA accuracy is optimised via accelerometers but there are implications relating to increased research costs and time which would need to be considered when designing a definitive trial [43,53]. Additionally, it would be most ideal to compare KAPA against a no-exercise control group to allow for the full effect of KAPA to be investigated.

Interventions are unlikely to be effective if recipients find components unacceptable [54].

Therefore, the KAPA intervention in its present form may benefit from being adapted using the participant perspectives prior to testing in a definitive trial [54].

## Conclusion

The KAPA intervention is feasible to deliver within community PA services. Attendance and retention rates were high suggesting KAPA is acceptable to attend. Overall, participants found KAPA acceptable, but the written materials would be better received if simplified. Illustrated home exercises and PA monitoring tools encouraged the participants to keep active. It is important for older adults exiting FaME programmes to remain physically active so as to maintain the positive health benefits gained. Therefore, a full-scale trial needs to recruit an adequate number of FaME classes to sufficiently power an RCT to assess whether KAPA results in a significant effect on maintaining PA.

## Data Availability

The datasets used and analysed during the current study are available from the corresponding author on reasonable request.

## Abbreviations

BCTs: Behaviour change techniques
CI: Confidence interval
FaME: Falls Management Exercise
FRAT: Falls Risk Assessment Tool
GBP: Great British pounds
ICC: Intra-class coefficient
IMD: Index of multiple deprivation
IQR: Interquartile range
KAPA: Keeping Adults Physically Active
MVPA: Moderate to vigorous physical activity
PA: Physical activity
PSIs: Postural stability instructors
